# “We really need to surround people with care:” a qualitative examination of service providers’ perspectives on barriers to HIV care in Manitoba, Canada

**DOI:** 10.1186/s12913-025-12514-1

**Published:** 2025-03-26

**Authors:** Cheryl Sobie, Katharina Maier, Margaret Haworth-Brockman, Enrique Villacis-Alvarez, Yoav Keynan, Zulma Vanessa Rueda

**Affiliations:** 1 Department of Medical Microbiology and Infectious Diseases, Rady Faculty of Health Sciences, University of Manitoba, Winnipeg, MB R3E 0J9 Canada; 2 Criminal Justice, The University of Winnipeg, Winnipeg, MB Canada; 3 Department of Community Health Sciences, Rady Faculty of Health Sciences, University of Manitoba, Winnipeg, MB R3E 0J9 Canada; 4 National Collaborating Centre for Infectious Diseases, Rady Faculty of Health Sciences, University of Manitoba, Winnipeg, MB R3E 0T5 Canada; 5 Department of Internal Medicine, Rady Faculty of Health Sciences, University of Manitoba, Winnipeg, MB R3E 0J9 Canada; 6 Facultad de Medicina, Universidad Pontificia Bolivariana, Medellin, Colombia

**Keywords:** HIV care, HIV service providers, Barriers, Qualitative research, Burnout, COVID-19, Canada

## Abstract

**Objective:**

To identify barriers to HIV care from the perspectives of HIV service providers in Manitoba (MB), Canada during the 2020–2022 period of the COVID-19 pandemic.

**Methods:**

In this qualitative study, we conducted semi-structured interviews with HIV service providers between October 2022 and January 2023. Purposive sampling was used to include a cross-section of 27 providers (clinicians, nurses, social workers, pharmacists, program managers, and health education facilitators). The main themes explored in the interviews included: (1) provider roles and organization; (2) facilitators and barriers to HIV care; (3) harm reduction and sexually transmitted and blood-borne infections prevention practices; (4) impacts of the COVID-19 pandemic on HIV care and providers and (5) policies related to HIV care in Manitoba.

**Results:**

Using a Social Ecological Model of Health framework, our analysis of service provider interviews identified barriers at four different levels: (1) structural level barriers, including limitations to public health and social support systems, geographic barriers, and policy inefficiencies; (2) socio-cultural/community level barriers, such as experiences of racism, stigma and discrimination leading to people living with HIV’s (PLHIV) reduced trust in the health care system; (3) institutional level barriers, which describe how lack of primary care for PLHIV, limitations to the HIV care delivery model in Manitoba, and system capacity limitations have created missed opportunities for linkage to HIV care; and (4) intrapersonal barriers that reflect how the interaction of structural, socio-cultural, and institutional level barriers challenge providers’ role performance and exacerbate risk of burnout and moral distress.

**Conclusions:**

Our findings demonstrate how multi-level barriers intersect to create challenges for both PLHIV and providers, limiting where and how people receive HIV care and impeding providers’ ability to perform their roles and provide effective, consistent HIV care. Given the key role of HIV providers in facilitating care, structural, social/community, and institutional changes are needed, as is further research to examine structural causes of burnout to develop meaningful interventions that support service providers’ mental health and well-being.

## Background

In 2021 and 2022, more people in Manitoba were diagnosed with Human Immunodeficiency virus (HIV) than ever previously recorded [1]. Our research found that Indigenous people, people experiencing houselessness, people who use drugs, and young females were overrepresented among those newly diagnosed with HIV [2]. Further, our data showed that 73.2% of females and 67.7% of males presenting for HIV care between 2018–2021 had at least one other sexually transmitted and blood-borne infection, such as syphilis, hepatitis C or gonorrhea [2]. Among people newly diagnosed with HIV between 2018 and 2021, 71.8% of females and 43.5% of males self-reported injection drug use, with the majority reporting methamphetamine use [2, 3]. The percentage of people diagnosed with HIV in Manitoba between 2018– 2021 who were experiencing houselessness at the time of diagnosis also increased; in 2018, 23.3% of females and 17.9% of males experienced houselessness, rising to 48.0% of females and 22.3% of males by 2021 [2]. These trends suggest that the convergence of methamphetamine use by injection, houselessness, and increasing socio-economic inequalities, have collectively increased people’s vulnerabilities in Manitoba, thus increasing their risk of acquiring HIV [3].

Life-long engagement in HIV care is required to ensure people living with HIV (PLHIV) maintain a good quality of life [4]. Concerningly, HIV treatment rates are lower among PLHIV experiencing houselessness [5] and those who use substances [6, 7]. Stigma and discrimination continue to contribute to incomplete or disjointed HIV care, especially for those facing intersecting stigmas as a result of HIV, drug use, and houselessness [8, 9]. In Manitoba, adherence to HIV treatment and engagement in care has been decreasing [2, 3], exacerbating risk of poor health outcomes and further transmission in the community [2, 10]. Limitations to healthcare access and quality of care in Manitoba are ongoing concerns for both providers and PLHIV. Since 2017, under the guise of “efficiency” and streamlining services, the Government of Manitoba closed urgent care centres [11–13], leaving individuals, and especially those with multiple disadvantages with fewer options for care. Simultaneously, staffing shortages and increased demand for services left healthcare workers bearing the brunt of an increasingly overwhelmed health system [11, 14–16]. These developments are concerning given that health providers’ ability to facilitate treatment [17] and provide support [18] has been shown to improve PLHIV’s access to and continuation in HIV treatment. Recent reports in Manitoba have documented the toll on healthcare workers from years of ongoing crises in the sector and then exacerbated by the coronavirus disease (COVID-19) pandemic [19–21]. Mirroring other Canadian provinces, a 2023 Doctors Manitoba Annual Physicians Survey found that 78% of physicians reported strain due to systemic/institutional issues (e.g., staffing shortages), such as feeling frustrated by system issues, administrative/red tape burden, and not feeling valued/burned out [20].

HIV care providers face several challenges that can result in personal and professional consequences [22, 23]. Burnout is among the most widely documented challenges [4, 17, 23–25]. Macks and Abrams show how organizational (e.g., resource limitations), individual (e.g., desire to make a difference), and contextual (e.g., HIV stigma) stressors all contribute to provider burnout [22]. Another study with 28 HIV providers across the U.S. reported experiences of racial discrimination and racial microaggressions outside of and within their workplaces as factors related to burnout [4]. In Tong et al.’s [26] study on sources of stress among healthcare providers who work with PLHIV in China, participants reported feeling stigmatized due to their roles, both within their workplaces and in their personal/social lives, and as a result noted relational challenges such as not being able to find a partner, and other medical staff avoiding contact with them [26].

HIV care providers have reported on institutional and organizational constraints that hinder care delivery [27, 28]. For example, providers in Ontario, Canada reported on resource limitations, such as not having enough time to develop strong relationships with clients, and due to inadequate staffing, going beyond their role capacity to meet clients’ needs [27]. Similarly, providers in Michigan, U.S. reported barriers, such as inefficient policies, long patient wait times, insufficient staffing to meet client needs, as well as pressure from administration to keep appointments short [28].

Other studies have shown that HIV care providers must navigate often complex ethical issues in their day-to-day work with clients [29, 30]. In Kaposy et al.’s ethnography of HIV clinics in Manitoba and Newfoundland and Labrador (Canada), such issues involved patient confidentiality, criminalization of HIV and non-disclosure laws, access to medication, and adherence to treatment [29]. HIV providers in their study experienced tension resulting from clinic and patients’ differing views on how to best ensure confidentiality and privacy for HIV clients [29]. The authors also note the lack of universal antiretroviral therapy (ART) medication coverage in Manitoba and Newfoundland and Labrador, and the conflict between knowing the burden for their clients who cannot afford deductibles for prescriptions while also recognizing the life-saving necessity of ART medication [29]. Further research has identified challenges facing HIV providers when trying to engage marginalized populations, such as older PLHIV who use substances [31], as well as how linguistic and cultural barriers affect health literacy and communication between PLHIV and providers [32]. While there are fewer reports of how structural barriers affect HIV care providers, a study that examined barriers and facilitators to care for street involved youth in Canada and Kenya found public policy, such as lack of government support for basic needs and lack of health insurance, impeded access to care for PLHIV, and limited community-based funding affected providers’ ability to reach and connect street involved youth with care and resources [33]. Several studies on providers’ perspectives on barriers to HIV care engagement [27, 29, 34, 35], intervention programs [36–39], and pilot programs [40, 41] further offer critical insights into the effectiveness of treatment modalities and engagement strategies, and how to address barriers to HIV care.

These existing studies highlight key issues at multiple levels (i.e., intrapersonal, interpersonal, social, institutional, and structural) that can affect both providers’ ability to deliver comprehensive HIV care, and health outcomes among PLHIV. We add to this body of work by examining service providers’ perspectives on barriers to HIV care and treatment in Manitoba and reporting on how these barriers interact at various levels result in challenges for both providers and PLHIV. Given their relationships with PLHIV, front-line providers are well positioned to understand how both structural and institutional constraints affect access to HIV care; they offer unique insights that can be leveraged to create change and improve health outcomes for PLHIV.

## Methods

This study reports on the second objective of a larger multi-method study that examines the gendered and intersectional circumstances in people’s lives that are leading to complex syndemics of HIV, syphilis, and other diseases in Manitoba. The first objective reported on data collected from the clinical charts of people diagnosed with HIV in Manitoba between 2018 and 2021 to present an epidemiological snapshot of the current state of HIV in Manitoba [3]. The second objective investigated the impacts of the COVID-19 pandemic on barriers to HIV care from the perspective of both PLHIV [42], and HIV service providers in Manitoba, Canada. This paper reports on our findings from interviews with service providers. The protocol for this research has been published elsewhere [43].

The study was approved by the University of Manitoba Health Ethics Research Board (HS25572; H2022:218), the First Nations Health and Social Secretariat of Manitoba, Nine Circles Community Health Centre, Shared Health Manitoba (SH2022:194) and 7th Street Health Access Centre.

This research was conducted using a collaborative community-based research approach, involving a multi-disciplinary research team, community organizations, people with lived experience, frontline workers, and a diverse Research Advisory Committee.

### Setting

This study was conducted with service providers in Winnipeg and Brandon, the two largest cities in Manitoba and site of the three Manitoba HIV program clinics, and in Swan River, a town in west-central Manitoba, Canada. Located in central Canada, the province of Manitoba spans nearly 650,00 square kilometers. Most of its population of approximately 1.4 million lives in the southern cities of Winnipeg (~ 900,000) and Brandon (~ 55,000) [44]. The remainder live in smaller cities, towns, and other communities, many of which are isolated in northern parts of the province.

People newly diagnosed with HIV or living with HIV are referred to the centralized Manitoba HIV Program, which then coordinates their care through one of their three clinic sites (two in Winnipeg, and one in Brandon). These sites also coordinate care for PLHIV in rural and northern regions.

### Participants

We used purposive [45] and convenience sampling [46] to recruit providers who reside in Manitoba and work with PLHIV from across Manitoba, including men, women and gender diverse persons, as well as from a range of racial/ethnic and cultural backgrounds. Recruitment procedures included contacting providers who work at the three Manitoba HIV Program care sites, requesting referrals from other providers, and contacting other health and social service organizations that serve PLHIV as well as people who use substances. Our aim was to include providers who offer direct services to PLHIV and managers or directors of programs for PLHIV and/or PLHIV who use substances. Forty service providers were contacted by email with requests to participate in an interview.

### Data collection

Data were collected by the research coordinator (CS) between October 2022 and January 2023. Participants were sent the consent form before the interview to sign and return, or they provided verbal consent at the beginning of the interview session. To accommodate participants’ schedules, all interviews were conducted virtually using Zoom or Microsoft Teams. Our semi-structured interview guide consisted of 19 questions. This interview guide was co-developed with people with lived experiences and has been published elsewhere [43]. Relevant to this article were the questions about: sex and gender, role, organization and services provided, facilitators and barriers to HIV care in PLHIV, barriers specifically for PLHIV who use substances, the impact of COVID-19 on clients, organization and providers’, types of supports provided for clients, creating safe environments, and HIV policies in Manitoba. Interviews were typically 45 to 60 min long, with some up to 90 min. Interviews were audio-recorded and some were video recorded. Video recordings were deleted after the session ended to ensure participant confidentiality.

### Framework to analyze barriers to HIV cascade of care

We categorize barriers to HIV care using a socio-ecological model (SEM) first introduced by Bronfenbrenner to describe how interactions and relationships between individuals and aspects of their environments guide human development [47–49]. Several researchers such as McLeroy et al. [14] and McClarty et al. [19] have since adapted this model for use in health promotion research. The SEM framework describes five levels for relationships and interactions: intrapersonal (e.g., personal characteristics and behaviours), interpersonal (e.g., relationships, social supports and networks), socio-cultural (e.g. relationships among institutions and cultural norms) institutional (e.g., within healthcare organizations), and structural (e.g., policies, governance and economics at all levels of authority) [50, 51].

### Data analysis

Using NVivo® 12 Pro qualitative data analysis software, we thematically analyzed the data [52, 53]. Thematic analysis is a method of qualitative data analysis whereby researchers identify and interpret concepts and themes within the data and then report on the patterns identified [52], often through a conceptual and reflexive model [53]. Braun and Clarke outlined a six-phase framework for thematic analysis that is widely used by researchers across disciplines [52]. We followed their framework beginning with transcribing the interview audio recordings, using Otter.ai and then the transcripts were reviewed for accuracy by CS and EV. Next, three research team members each coded an initial subset of transcripts (n = 4) to identify initial codes and draft a preliminary codebook. After discussion and comparison of the subset of transcripts and initial coding, we refined the codebook which CS used to code the remaining transcripts. CS, KM, and ZVR jointly reviewed the codes and final analytical output. We grouped the data into five overarching categories: (1) Barriers to HIV care; (2) Facilitators to HIV care; (3) Impact of the COVID-19 Pandemic; (4) Recommendations to improve HIV care; and (5) Knowledge Translation Strategies. Within the first four categories, we identified multiple level factors that aligned with the SEM framework: Individual, Social/Cultural, Structural, and Institutional/Organizational. The data are presented in aggregate form in this paper, across participant roles and personal identities.

## Results

**Fig. 1:**
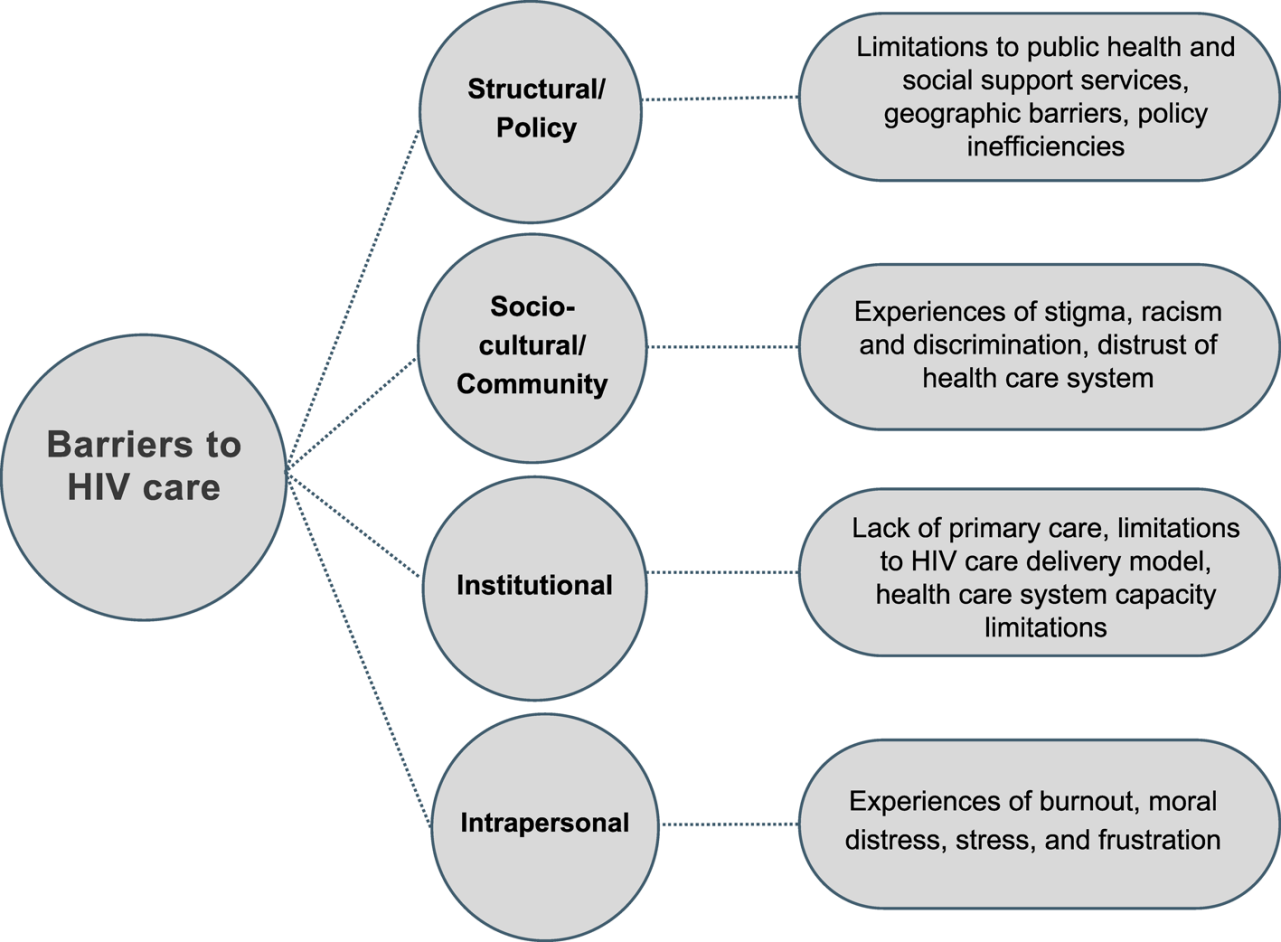
Barriers to HIV care depicted using the Social Ecological Model (SEM) (adapted from McClarty et al. [49]

Twenty-seven providers participated in the interviews. Participants held a variety of roles (Table 1) providing support or treatment for PLHIV, including from public health and programs as well as harm reduction services, and identified as women, men, and non-binary. Figure 1 depicts some of the barriers to access HIV care that our research found.

**Table 1:** Participant roles providing services to people living with HIV in Manitoba

Role of Service Provider:	Number of Participants (*n* = 27)
Pharmacist	1
Health Education Facilitator	1
Community Health Nurse	2
Nurse Clinician	2
Social Worker	2
Public Health Nurse	4
Nurse Practitioner	5
Program Manager or Director	5
Physician/Clinician	5

### Structural: geographic barriers, limitations to public health and social services, and policy inefficiencies

Providers reported several structural barriers: geographic barriers; limitations to public health and social support systems; lack of comprehensive health services (e.g. mental health and substance use treatment services); and policy inefficiencies.

Participants spoke positively about many aspects of Manitoba’s HIV Program sites, such as that these clinics offer both routine HIV health services as well as more specialized health services involving dieticians, psychiatrists, and other mental health professionals, and social workers. Referrals to other professionals are made as needed. Participants noted that while this model generally works well for PLHIV in Winnipeg and Brandon, the same level of care and support is not readily available for PLHIV in smaller and more remote communities, mainly in northern Manitoba. Virtual care options are in place so that PLHIV can connect with providers via telehealth, facilitated by staff at a rural or northern nursing station. Providers may also arrange travel to Winnipeg for PLHIV requiring specialized care. However, transportation is difficult for anyone without a personal car and there are few public transportation options for people from remote communities (there are no public buses, for example), leaving many unable to get care in person. Thus, Manitoba’s centralized HIV care system, while providing benefits to some, currently prevents PLHIV in rural and northern communities from receiving the same level of care:

…*The centralized or specialist model is, is a bit of a barrier. So, telehealth…the way we were utilizing it, works okay for some folks, I would say a minority of our HIV positive population. But they need the services when they need it, not when we can schedule it for them…so some of that is a challenge. So, transportation is a huge [barrier], marginalization of folks that distance to travel…within communities.* (Participant 13)

Additionally, participants noted how staffing challenges at nursing stations in rural and northern communities make it difficult for providers to connect PLHIV to care. For example, Participant 1 explained how staffing shortage and turnover can result in care being routinely delayed and fragmented:

*It’s very difficult to contact anybody at a nursing station on a good day. And then even if you do reach someone… that person's only there for a week. And then there's turnover. And I know the doctor is not here until next week, and so... the care is so fragmented, even if the patient is motivated to get care… there are some communities that…. have a huge struggle connecting with patients and often never get people into care or on medication....* (Participant 1)

While the lack of care is most acute in rural and remote communities, PLHIV in urban settings also face challenges to receiving care. PLHIV who experience additional forms of marginalization (e.g., houselessness) may also have no transportation (e.g., lack of personal vehicle, limited public transit options), making it difficult to get to and from appointments:

*We expect people to, oh yeah you can drive, you can move, you can do all these things. It's easy for the folks who make up these plans…But when you've never lived on the street, you don't know what it's like to have to take a bus, with poor bus service…how do you safely get to the hospital using public transportation?* (Participant 21)

Providers explained that *“it's the big systemic underlying social factors that are becoming the biggest barrier to access care”* (Participant 12). They described how PLHIV face many, often compounding, structural barriers such as poverty, houselessness, and insufficient supports for substance dependence and mental health challenges, which exacerbate challenges in access to and engagement in HIV care. Several providers voiced concern about the lack of publicly funded mental health and substance use treatment options:

*I think in a system where mental health…supports for people who are suffering, emotionally, mentally, are very strained, treatment programs where they exist, are very heavy with very limited bed space, let alone treatment programs that reflect cultural views that are honoring the people that are in that program.* (Participant 26)

The lack of comprehensive mental health and addiction treatment is problematic as research shows access to such services can increase PLHIV’s likelihood to connect and stay engaged with HIV treatment [36]. As Participant 6 explained, provision of HIV medication and care is only effective when patients’ “*root problems*” (e.g., mental health, addiction) are addressed concurrently:

*It's ridiculous…we can do all these other things, we can give you medication I can put you on, you can do this. But again, the root problem we know… people need talk therapy, they need to get through their trauma. And that is something that seems unattainable for, I would say, 99.9% of the population I deal with.* (Participant 6)

In addition to mental health and addiction, the challenges associated with being houseless create barriers to HIV treatment. According to participants, PLHIV experiencing houselessness and/or using substances focus on their daily survival (e.g., where to sleep, eat, how to stay safe) which means they may not be able to prioritize their health needs. As one participant stated:

*Homelessness, that's huge. Because…HIV is on the lower list of important things. When you don't have a home, you don't have food. You know, those are priorities…if somebody's using, their priority is substance [use] right? When they're going to get their next substance, where they're going to be sleeping that night?... Those are priorities in folks’ lives usually over health care…* (Participant 11)

Furthermore, providers noted it is particularly difficult to get in touch with clients who do not have a fixed address or phone:

*…just being able to call and talk to somebody in the moment, because so many people don't have phones, or, you know, they're on minutes and everything is really, you know, limited.* (Participant 16).

PLHIV staying in a shelter or sleeping rough face additional hurdles, such as having no place to securely store their medications. Providers commented that this often means medications are stolen, leading to gaps in treatment:

…*because they don't have a safe place to keep their medications, we run into the problem where people get their meds stolen or lost. And then it's really hard to get those medications replaced. And so, many times people are without, even though they would be willing to take the medications on a daily basis, they just don't have it.* (Participant 10)

Participants talked about three key policy inefficiencies that affect PLHIV’s engagement in treatment and hinder providers’ ability to connect PLHIV with critical services and treatment: limited ability to offer harm reduction services; lack of universal HIV medication coverage; and lack of standard procedures for sharing information.

Most providers described how the lack of universal coverage for HIV anti-retroviral medications (ART) and pre-exposure prophylaxis (PrEP) in Manitoba created challenges for both PLHIV and providers. Participants explained that PLHIV could not afford to pay for ART medications without some sort of insurance or health benefits (such as those provided by an employer) and many do not register with Pharmacare, the subsidized drug program in Manitoba. The federal Non-Insured Health Benefits program does pay for HIV medication for PLHIV who identify as First Nations or Inuit, however navigating the necessary systems requires a great deal of time for clinic staff, especially pharmacists.

*You know, some people don't even want to start medication or connect with care, because they've heard how expensive medication is. And so, there's a barrier there, even before we get a chance to see them. We always work through it. You know our pharmacists are very good at finding a way but not having universal coverage is probably the number one issue that we deal with, on a daily basis.* (Participant 1)

Providers also noted that harm reduction services are key to facilitating HIV care for PLHIV who use substances, and especially for those who are disengaged or inconsistently engaged with care. However, harm reduction supplies and support are not consistently available in Manitoba. To illustrate, several participants noted that hospital policy sometimes prevented HIV clinic staff from providing PHLIV with critical harm reduction supplies:

*And we don't really have a harm reduction [policy], per se. And that's an [institutional] policy that complicates things. Well, obviously, we talk about harm reduction, it’s just that we can't get supplies unless we sneak them to the patient.* (Participant 27)

Another provider explains how the lack of a harm reduction policy affects client safety:

*So, if somebody's you know, we can give them a few [needles]…But it's just that we're not a harm reduction facility. We don't even have Naloxone kits. They might in emerge [emergency department]. I don't know, but we don't have them in the clinic, which I've brought up in the past, which I think is crazy.* (Participant 3)

Participants noted the importance of ‘wedding’ HIV with harm reduction care, as offering harm reduction supplies to PLHIV who use substances can be an “*incentive*” for PLHIV to access HIV care:

*And I think that's* [provision of harm reduction supplies] *a huge draw and would be a huge addition to our clinic. And I think it would make it more inclusive, and it would also provide…some incentive for people to connect with us.* (Participant 1)

Service providers’ ability to provide harm reduction and other services to PLHIV necessitates inter-agency collaboration and information sharing across service providers. However, several participants noted the lack of a standardized charting system or database that would allow them to easily find and retrieve client information. Not only is this time-consuming, but, as Participant 8 explains, the lack of information-sharing and centralized coordination can lead to delays or missed opportunities to link PLHIV to care at the time of diagnosis:

*So, a big policy gap is around information sharing around client information. So technically, the HIV program does not have access to patients who are diagnosed with HIV until…they're referred to the program. And what that, unfortunately, enables is this real inefficiency, from time to diagnosis to linkage to care. So, if the policy that we're working towards is getting access to client information at the time of diagnosis so we can work with public health...to quickly link folks to care.* (Participant 8)

These structural barriers demonstrate the disconnect between the ‘high level’ decisions of where and what types of services are available (i.e. geographic availability, limited harm reduction policies, lack of substance use treatment resources) and the real-world consequences that result in continually limited access to comprehensive HIV treatment, harm reduction services, and social supports for marginalized PLHIV.

### Socio-cultural/community: experiences of racism, stigma, discrimination and distrust of health care system

Participants reported that clients experience routine racism and discrimination in the healthcare system, based on personal observations and listening to their clients recounting such experiences. Participants explained how discrimination and negative experiences with the healthcare system create barriers to engagement in care for PLHIV:

*Okay, so racism?…a lot of my clients are First Nations people; they don't trust the health care system for good reasons. There's been generations of discrimination against them. If they go into…say to the emergency departments, they're discriminated against, so why would you want to go and connect with these healthcare providers that are going to judge you?…they don't trust the health care system. They feel judged...* (Participant 9)

Providers described how they work to mitigate some of these barriers through a non-judgmental approach to care provision, working to build safety and trust with their clients, reflecting on their own assumptions and biases, and in some cases calling out the biases they notice among their colleagues and organizations. However, as one participant noted, some patients’ distrust in the health system is so severe that they would rather endure negative health consequences than seek institutional help. Recounting one client’s experience, Participant 6 explained:

*One gentleman has a hole in his arm that goes almost right down to his bone. And it started as a small wound that I couldn't get him care for. I've had to redirect him back to the emergency room. At this point he needs plastics, he needs IV outpatient antibiotics. But I continue to question what could have been done if we had early intervention with that? And it's also, very telling of the state of the healthcare system, and how people who use drugs feel accessing the system, because he would rather lose an arm than go sit in the emergency room or stay in hospital for care. So, it’s really sad.” *(Participant 6)

These participants’ quotes must be contextualized within broader histories and contemporary colonial violence affecting Indigenous people in both Manitoba and across Canada. It is not surprising participants noted racism, stigma, and broader socio-economic inequities as major barriers to HIV care, given research has consistently shows that Indigenous people are underserved in healthcare and social institutions [54–56], and that Indigenous peoples’ experiences of racism and discrimination within the Canadian healthcare system negatively affect health outcomes [57–59].

There are similar challenges in an emergency department, as one participant commented:

*There’s a lot of barriers and then there's stigma within the emergency department. When people come in, even though there's, I want to say, 99%...an underlying psychiatric illness…they get labeled as meth induced psychosis. And so, they never get seen for the underlying psychiatric [issue]. I have a [client] right now, for sure I feel she's schizophrenic. But she keeps getting labeled. And so, we get nowhere, because oh, she uses meth. And so that's, I feel, a cop out that they use meth. And so now we can't [treat them]... there's no plan to, there's no treatment that is really imminent and long term.* (Participant 3)

Given many PLHIV’s first point of contact is with emergency department physicians and/or nurses, negative and stigmatizing experiences with emergency staff can create a ‘ripple’ effect’, meaning clients may be discouraged from seeking further care in community spaces because of negative interactions at the emergency department.

Providers felt that their organizations were doing their best to provide safe, welcoming, and inclusive spaces for their clients, but they also highlighted the need to adopt additional culturally safe approaches such as having an Indigenous Elder and other spiritual or cultural support available during clinic hours.

*I think having things that are not culturally safe, not culturally appropriate, not culturally relevant, that do not have cultural supports built in…is a barrier. And beyond First Nations populations here, we also have a lot of immigrants that are coming. That language alone is a giant barrier...we’re far behind on that.* (Participant 6)

Further, while some workplaces were reported to have a mix of diverse gender, sexual, and racial identities among staff, several other participants noted a lack of racial/ethnic diversity in their workplaces. They commented that their organizations actively seek more diverse team members when hiring, and they also recognize the effect the lack of representation from certain populations may have on their relationships with clients. Providers also described their organizational and individual efforts to take part in anti-oppression and inclusivity training and cultural learning opportunities, although there are limitations to how much can be done during work hours, and providers reported needing to complete training on their personal time.

### Institutional: lack of primary care, limitations to hiv care delivery model, and health care system capacity limitations

Providers noted that many PLHIV do not have a primary care provider. While two Manitoba HIV Program clinics offer primary care services beyond HIV care, participants explained that in other cases HIV nurse practitioners or clinicians must step in to provide routine health checks, such as Pap smear tests or vaccinations. While this helps clients with their immediate needs, it is not sustainable and can lead to an overwhelming workload. Participant 1 explains:

*The clinic is meant to provide specialized HIV care. But in reality, that is not often the case. There's quite a number of patients who don't have solid primary care. And so, there's a lot of additional responsibilities that people are taking on [that] clinicians are having to address when people present for care.* (Participant 1)

Other participants noted a lack of capacity and training among primary care providers to manage HIV care. With the advances in medications and treatment, many PLHIV could have their care managed by another primary care provider, which would help to alleviate some of the pressures HIV clinics face with the increasing number of HIV diagnoses.

Participants explained that many organizational aspects of the current HIV care delivery model and the broader health care system are disconnected from PLHIV’s lives. For example, as Participant 2 noted, for people with *“addictions”, “histories of trauma, histories of abuse…”,* fixed clinic hours and set appointment times can be difficult to manage. Participant 22 echoed these concerns and Participant 24 noted the need for flexible clinic hours and schedules:

*“Oftentimes, with the clients I'm thinking of, they're still actively using, so it's really hardfor them to remember* [an appointment]*, then they'll randomly show up, and they want tostart treatment, but…now we don't have an HIV clinic for a while coming up...”.*(Participant 22)

*I think with the appointments they'd be better if we could do later in the evening…Because daytime appointments are great for the staff… but then you're missing some of the clients that are in most need to get connected to care…. And I think that…also sets people up for failure, because we'll be like, we're open till five, but you have to come at four*” (Participant 24)

Many providers explained that due to shifting needs of newly diagnosed PLHIV, a more “*in the moment*” approach to care delivery is necessary, such as more street-based nursing, extended availability of mobile services, or allotting space for walk-in appointments. Participant 21 describes this as, “*meeting people where they're at…finding people where they're at and trying to provide services for them, whatever they feel it is that they need at that time.”*

These providers stressed that a more complete shift to *“in the moment”* care can only happen with an influx of resources such as funding for additional staff positions and for more outreach services. Participants explained that while the Manitoba healthcare system had capacity issues before the COVID-19 pandemic, the combination of the increasing number of newly diagnosed PLHIV and clients with more complex health needs has created an increased demand for services. Several participants reported not having enough staff and increased workloads because positions are vacant: *Same thing with our nurse practitioner, which is great, because if they don't have any kind of primary care, she kind of takes them on as we get those new referrals. However, her workload is enormous and we're down a couple of providers here too. So, I feel like once again, it's the one to two weeks period before we can try to get some of our clients in and it's becoming an issue* (Participant 12).

The lack of primary care, inaccessibility of HIV clinics in rural and remote areas, and capacity limitations mean some PLHIV seek care in settings that are not set up for HIV and primary care. Providers observed a shift over the previous three years (2019–2022) to more PLHIV seeking care at hospital emergency departments. Participants mentioned several reasons for this: their location is more central, emergency departments are open all hours, there are increased numbers of PLHIV who are houseless and have no means of transportation or a primary care provider, and in some cases people need a safe place to sleep or wait out substance use intoxication. Participants reported that PLHIV may present in an emergency department with co-morbidities but the opportunity to connect them with HIV-specific care is often missed. Providers noted this may be due to emergency department staff having too little time or knowledge to support someone with a new HIV diagnosis and to arrange follow-up care. Participant 21 explains the knock-on effects of limited support for one of their clients living with HIV:

*“I'm working with someone who says, “I have been HIV positive for a year”, she just continues to go to emergency departments. And they write on [her charts], oh, she's not following up. And I asked her, “why aren't you following up?” And she's like, “where am I supposed to go? Nobody tells me. I'm supposed to go to [clinic]? I don't have any appointments.” And if they do…she wasn't aware of those…And doesn't have access to computers, doesn't have access to a phone, living on the streets, homeless. So, we really need to surround people with care instead of, oh, they'll figure it out themselves.* (Participant 21)

Several other participants noted how critical it was to link people to care at the time of diagnosis to ensure immediate treatment. As Participant 25 noted: *“…we know that once somebody is connected with care using that opportunity to set the path of engagement on a different course.”* (Participant 25). However, if this does not happen, particularly for clients experiencing houselessness, it is incredibly challenging for providers to locate them after the fact, creating more work for providers and resulting in gaps in treatment for PLHIV.

### Intrapersonal: experiences of stress, burnout, moral distress and frustration

Providers described the past three years as incredibly challenging and reported personal experiences of increased stress, burnout, moral distress, frustration, and exhaustion. They recognize these experiences result from working with people with numerous disadvantages, while being unable to facilitate the care their clients need. Several participants noted that while these stressors existed prior to the COVID-19 pandemic, they were amplified due to the increased demands on health services.

*I think there's more burnout now… nobody's really had a break, we've gotten accustomed to a much higher level of terribleness of everything… and then also…moral distress of knowing how things used to be, knowing that people didn't use to have to wait long for a procedure… to be seen in emerg… to wait that long for a bed… the moral distress that comes with knowing that what's happening now is not normal.* (Participant 23)

As many providers had been in their roles for more than three years, they were frustrated by the continual decline of social supports, increasing houselessness, and substance dependence, all exacerbated by COVID-19, that affected their clients’ health and well-being. Despite their best efforts to provide care, providers noted there are many needs that go unmet. Participant 9 explains:

*...We need a whole provincial government response, and we're not getting that… because it's people that are homeless, and its people who use drugs. So, the government doesn't give a shit… I will stand by that. It's racist. It's really challenging and frustrating to be a public health nurse when you see so much needs to be done, and it's not happening.* (Participant 9)

While the participants described their passion for their work and their relationships with clients as protective factors, most also described situations of being pushed beyond the capacity of their roles. As a result, some participants reported a decline in their own health and wellbeing, a desire to leave their roles and retire as soon as possible, or feeling they are merely responding to crisis after crisis instead of delivering care within their scope of practice.

*I feel like you’re kind of on your own... you're just doing damage control every day. And you can't make any real [pause] because there's so many patients and they're all in a state of crisis, and… you're doing things to keep them alive every day… [sigh, pause]...I don't know. It's just, it’s crisis management is basically what it is. Because there's no time when you have so many patients and to do real in-depth stuff.* (Participant 3)

Many other providers echoed similar sentiments, demonstrating that there are multiple daily stressors, largely driven by factors outside of providers’ control that are contributing to their experiences of stress, frustration, burnout and moral distress.

## Discussion

Drawing upon interviews with HIV service providers, we report on the numerous converging factors that created barriers to HIV care and treatment in Manitoba during a specific period (2019—2022). We find that providers’ understanding and experiences of barriers to care operate at structural, socio-cultural, institutional and intrapersonal levels. Our findings suggest the need for both healthcare and policy changes that could improve efficiency and access to care for PLHIV, while at the same time, supporting providers to deliver high-quality care and preventing burnout. Studies in British Columbia, Canada, have shown that the province’s institution of universal coverage of drugs and other health-care costs for PLHIV in 1996, has increased cost-effectiveness [60], decreased mortality and morbidity among PLHIV [60, 61] and decreased HIV incidence [61]. In 2024, the Manitoba Government began the Manitoba HIV Medications Program (MHMP) [62, 63], which covers the cost of most HIV medications (including ART’s and PrEP) [62]. However, concerns remain that individuals still have to bear the costs of medications for common comorbidities, shifting the problem to other chronic conditions (such as diabetes, etc.). It will be important to assess in the coming years what effect this policy change may have on PLHIV’s ability to consistently remain on ART treatment and if there is a corresponding improvement in workflow and efficiency for providers.

Our findings show that the interaction of barriers at three different levels (structural, institutional and socio-cultural) meant that, according to providers, some PLHIV avoided seeking care or were unable to get to care, leading to them becoming sicker and requiring more complex care, often presenting in a state of ‘crisis’. Providers attempt to address as many of their clients’ health needs as possible, even those that other health institutions/actors might be better equipped to address; however, their capacity is limited. Failure to properly resource HIV care is indicative of systemic failure by governments to address underfunding of health and HIV care in Canada [64, 65] that has resulted in unmet mental health care needs [66, 67]; drug poisoning crises that are disproportionately affecting Indigenous communities [68, 69]; and out-of-pocket expenses that strain social welfare incomes below the poverty line [70].

Participants noted that for many of their clients, long-term and consistent engagement in care may only be possible if PLHIV’s basic needs are met (e.g., adequate income, food, shelter), and if they can obtain other critical health services such as treatment for substance use and mental health supports. In a systematic review of barriers and facilitators for HIV care among migrants in Organization for Economic Co-operation and Development (OECD) countries, Arora et al. similarly found that unmet basic needs were critical impediments to consistent HIV care [71]. Studies have shown the effectiveness of culturally responsive mental health treatment integrated with HIV care, such as in New York, USA, with participants reporting improved mental health and decreased HIV-related symptoms [70], and in Cape Town, South Africa, participants reported decreased depressive symptoms and improved ART adherence [72]. Similarly, other studies report on the effectiveness of harm reduction strategies in supporting more consistent ART use through directly administered antiretroviral therapy (DAART) from a mobile health care van in New Haven, Connecticut [73], and a peer-led telehealth harm reduction intervention [74] among PLHIV who use injection drugs in Miami, Florida.

Participants spoke about the necessity of offering harm reduction services in conjunction with HIV care, as not only an avenue to better support engagement and retention in HIV care, but as critical component of healthcare, given the increasing number of drug poisoning deaths in Manitoba [75–77]. The consequences of having an HIV clinic in a major healthcare centre that lacks a centralized harm reduction policy is problematic for providers. “Sneaking” supplies to clients may lead to unsafe situations where clients choose to re-use supplies. Further evidence has shown a sharp increase in hospitalizations [78] and excess mortality due to substance use in Canada since the start of the COVID-19 pandemic [79, 80], highlighting the urgent need for policy changes at both institutional and structural levels. One positive outcome in Manitoba has been a 2024 commitment by the provincial government to fund the Aboriginal Health and Wellness Centre in Winnipeg to implement mobile HIV services as well as a partnership to develop the province’s first Indigenous-led safer consumption site [81].

Our examination of barriers to HIV care suggests that racism and discrimination continue to impede Indigenous people’s access to healthcare in Manitoba. Indigenous people’s experiences of discrimination in the Canadian healthcare system have been documented in the attitudes and practices of care providers [82, 83] and reports reveal how racism has led to devastating health outcomes for Indigenous people in Canada [84, 85]. For example, Woodgate et al. examined Indigenous PLHIV’s experiences of stigma and discrimination in Manitoba in which a woman recounted interactions with the child welfare system which resulted in her child being apprehended at birth, simply because she was HIV-positive [86].

A recent comprehensive study of Indigenous females' healthcare in Canada during reproductive ages (15–55) found disparities in access to primary care and unmet health needs when compared to non-Indigenous females; and after sociodemographic adjustment, the findings suggest these disparities are the result of systemic racism and mistrust of the healthcare system [87]. The authors note this highlights issues of both limited access to primary care and a lack of culturally safe and trauma-informed primary care providers and recommend improvements that are aligned with healthcare reconciliation [87]. Similarly, as noted by providers in our study, systemic racism and experiences of discrimination results in both access challenges as well as disjointed care due to mistrust of the healthcare system and providers.

Participants proposed recommendations to address bias and discrimination and increase cultural safety such as more organizational support for staff anti-racism and cultural safety training, incorporating more cultural/spiritual care into their clinics, hiring staff reflective of the clients they serve, increasing access to and capacity of primary care providers, and answering the calls of Indigenous service delivery organizations to ‘act now’ to improve the health and wellbeing of Indigenous communities. The recommendations providers proposed align with the Truth and Reconciliation Commission of Canada’s Calls to Action to improve the experiences of health among Indigenous peoples in Canada. Seven of the 94 calls to action (calls 18–25) are specifically focused on health and healthcare [88, 89]. Relevant to our study are the calls to action that urge the Government of Canada to close the gaps in health outcomes between Indigenous and non-Indigenous people and publish annual indicator reports including for chronic diseases; recognition of Indigenous healing practices and ensuring access to Indigenous Elders, ceremonies and medicines; increasing the number of Indigenous professionals in healthcare; and ensuring cultural competency (i.e. cultural safety) training for all healthcare professionals [88, 89].

Given that HIV care providers in Manitoba are at the front-lines of providing care, they experience the brunt of structural and institutional failures, resulting in personal and occupational consequences, such as increased stress, experiences of burnout and moral distress, and in some cases, working outside of their scope of practice. Studies on burnout among healthcare workers have recommended individual or institutional and organizational level interventions [90, 91], such as resilience or mindfulness training [92, 93], or to address workload [94] and staffing shortages [95]. Similarly, studies addressing burnout among HIV care providers highlight interventions such as improving organizational culture [24] and communication [22, 25], or increasing psychological resources [4].

Affleck and Wagner argue that most Canadian studies of burnout focus on institutional-level factors such as workload, institutional culture, and time pressure, while failing to examine structural factors, which they define as “*federal, provincial and territorial governance, legislative, regulatory, fiscal, strategic and cultural approaches to health care sector oversight and management”* [65]. They note there are fewer studies that highlight the causal relationship between system-level and structural factors [96, 97] and healthcare provider burnout, suggesting this may be due to the consequences of service-level decisions that affect health care workers day-to-day roles, such as health policy, hiring practices, capital investments, and governance decisions, can often take years to become evident, which can mask the causal link between system level factors and burnout [65]. Rather, Affleck and Wagner suggest that in Canada it is the structural determinants of burnout that are responsible for healthcare worker burnout, and a better approach would be to examine systemic and structural level burnout factors to formulate more effective interventions [65]. Similarly, a recent paper from the Ontario Medical Association has reported on system level factors as root level causes of burnout among physicians (and other health care professionals), that require system level solutions such as ensuring fair and equitable compensation and organizational policy changes [98]. This evidence aligns with what healthcare providers in our study have said;, structural changes such as policy changes, increased healthcare sector resources, increased governmental support for PLHIV to meet their basic needs, and improved functioning of the healthcare system, would likely bring the largest improvements for client care and provider health and well-being.

Our findings suggest that many of the barriers described by providers existed before COVID-19 and were exacerbated as a result of the pandemic. Our team will present more comprehensive findings on the effects of the COVID-19 pandemic on HIV care and service providers in Manitoba, in a complementary paper (Manuscript in preparation, 2024).

### Future implications

Based on our findings that (1) numerous barriers continue to impede access to HIV care and HIV care provision; and (2) HIV service providers are experiencing high levels of burnout and moral distress, we propose the following next steps within the field of HIV care research: (1) *Develop and implement adaptive HIV care provision strategies to meet the needs of changing populations; (2) Integrate HIV care with other services such as substance use treatment, mental health services, other health and social services;(3) Advance cultural safety and health care reconciliation at system level; (4)Examine burnout among health care providers at structural level and design appropriate interventions; (5) Invest in HIV prevention and harm reduction.*

Another important consideration for governmental and institutional decision makers raised by participants in our study was: How will healthcare systems and providers cope with high number of people living with HIV in Manitoba who are not receiving treatment and are not consistently engaged in HIV care? Further, our interviews revealed important data about how providers are reshaping their roles to adapt to and navigate these complex and changing circumstances.

### Limitations

This study has several limitations. First, we used purposive and convenience sampling to recruit participants, aiming to reach a diverse group of HIV providers in Manitoba. Given time constraints, several key informants that we hoped to interview declined to participate or did not respond to our requests for an interview. Second, while the sample size (n = 27) yielded rich descriptive data, Manitoba’s unique situation (rapidly increasing HIV incidence) means that our findings may not be generalizable across contexts. However, they may be transferable in locales with similar contextually relevant factors [99]. Third, the providers we interviewed work primarily in Winnipeg and Brandon, Manitoba, with only one provider residing in a northern community. We anticipate that interviewing providers in rural and northern communities would highlight additional and potentially different barriers for both PLHIV and providers.

## Conclusions

Our findings offer relevant insights to HIV healthcare provision in a high-income setting. Given that HIV diagnoses are increasing in other countries [69–71], we encourage researchers to examine how social and structural contexts in their own jurisdictions may influence provider barriers. There are several findings that are of relevance to other contexts, including how increasing substance use, socioeconomic marginalization, and experiences of discrimination impede consistent engagement in HIV treatment, necessitating the need for adaptive engagement strategies.

We found that institutional, structural, and socio-cultural level barriers are connected and reinforce each other, limiting where and how people receive HIV care and impeding providers’ ability to provide effective, consistent HIV care. For example, it is likely that at least some of the barriers reported by participants would be decreased if the healthcare system were better resourced and structured, if providers had more manageable workloads and there were policies that enabled consistent care.

Finally, while HIV service providers play a critical role in supporting PLHIV to maintain a good quality of life, burnout among care providers is a growing concern that has serious implications for both healthcare system users and institutions. As recent studies have identified, both structural [65] and institutional/organizational issues [100] appear to play a significant role leading healthcare providers to experience burnout and in some cases to consider leaving their roles [94, 100–102]. The COVID-19 pandemic has exacerbated existing challenges [25, 67], which has resulted in worsening mental health and experiences of burnout among healthcare providers [103, 104], prompting renewed calls for system-level changes to improve provider well-being [94, 105, 106]. The burden of trying to meet increased demands for service without new resources and other policy changes cannot continue to be placed on the shoulders of care providers. Therefore, it is imperative to change the culture from one where burnout and moral distress are normalized, to one that recognizes burnout and moral distress as system failures and it is simply unacceptable to ignore the calls to action from frontline providers. Actions by advocacy groups such as the Manitoba Nurses Union provide promising examples of how change can be achieved [107, 108]. While there is more to be done to improve the health care sector in Manitoba, this is one step that may pave the way for future positive change for PLHIV and care providers.

## Data Availability

Individual participant data will not be available since it contains potential identifiable information.

## Abbreviations

ART: Antiretroviral therapy
COVID-19: Coronavirus disease
HIV: Human Immunodeficiency Virus
PLHIV: People Living with Human Immunodeficiency Virus
PrEP: Pre-exposure prophylaxis
SEM: Socio-Ecological Model
